# Ras inhibitors gate chemoattractant concentration range for chemotaxis through controlling GPCR-mediated adaptation and cell sensitivity

**DOI:** 10.3389/fimmu.2022.1020117

**Published:** 2022-10-20

**Authors:** Xuehua Xu, Tian Jin

**Affiliations:** Chemotaxis Signaling Section, Laboratory of Immunogenetics, National Institute of Allergy and Infectious Diseases, National Institutes of Health (NIAID/NIH), Rockville, MD, United States

**Keywords:** chemotaxis, G protein-coupled receptors (GPCRs), adaptation, Ras, guanine nucleotide exchange factors (GEFs), GTPase-activating proteins (GAPs), *Dictyostelium discoideum*, neutrophils

## Abstract

Chemotaxis plays an essential role in recruitment of leukocytes to sites of inflammation. Eukaryotic cells sense chemoattractant with G protein-coupled receptors (GPCRs) and chemotax toward gradients with an enormous concentration range through adaptation. Cells in adaptation no longer respond to the present stimulus but remain sensitive to stronger stimuli. Thus, adaptation provides a fundamental strategy for eukaryotic cells to chemotax through a gradient. Ras activation is the first step in the chemosensing GPCR signaling pathways that displays a transient activation behavior in both model organism *Dictyostelium discoideum* and mammalian neutrophils. Recently, it has been revealed that C2GAP1 and CAPRI control the GPCR-mediated adaptation in *D. discoideum* and human neutrophils, respectively. More importantly, both Ras inhibitors regulate the sensitivity of the cells. These findings suggest an evolutionarily conserved molecular mechanism by which eukaryotic cells gate concentration range of chemoattractants for chemotaxis.

## Introduction

Chemotaxis is directional cell migration guided by extracellular chemoattractant gradients. This behavior of eukaryotic cells plays critical roles in many physiological and pathological processes, such as recruitment of leukocytes to sites of inflammation, metastasis of cancer cells, and the early development of the model organism *Dictyostelium discoideum* (1–3). Mammalian neutrophils and *D. discoideum* use G protein-coupled receptors (GPCRs) and GPCR-mediated signaling pathways to sense and respond to chemoattractant gradients for chemotaxis (4, 5). One key feature of eukaryotic cell chemotaxis is that a cell is able to sense and translate extremely shallow chemoattractant gradients, as little as a 2% difference in chemoattractant concentration between the front and the back of a migrating cell, into polarized intracellular responses, a process named signal amplification (6–10). Another fascinating feature is that cells chemotax through gradients with an enormous concentration range (10^-9^ to 10^-5^ M cAMP in *D. discoideum* and 10^-9^ to 10^-5^ M fMLP or IL8 in neutrophils) (11, 12). To achieve the above two features, eukaryotic cells employ a strategy called adaptation (13). Adaptive cells no longer respond to the continuing, existing stimulus but remain responsive to stronger stimuli. Adaptation is believed to provide a fundamental mechanism for gradient sensing and directional cell migration toward the source of chemoattractant gradients with a huge concentration range (13, 14).

**Figure 1:**
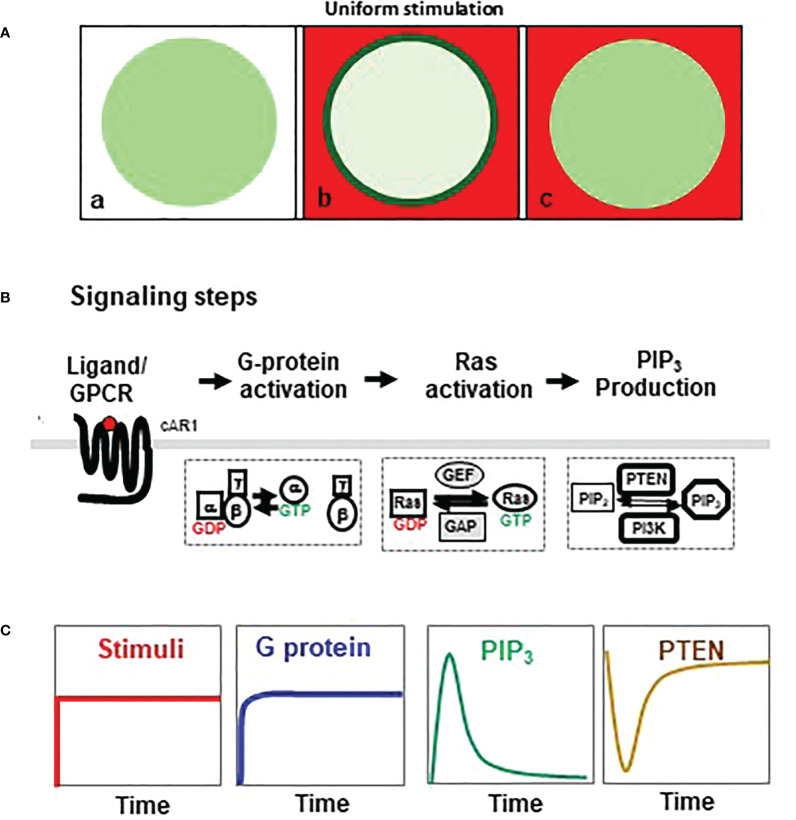
Adaptative activation of heterotrimeric G protein, Ras, and PI_3_K. **(A)** Homogeneouly applied (uniform) stimulation triggers a transient membrane translocation of a cytosolic protein in the cells. a, a resting cells expressing a cytosolic protein (green); b, cell is stimulated by uniform stimulation (red) and displays a response as membrane translocation of cytosolic protein to the plasma membrane (green); c, cell in adaptation, in which the cell’s response is terminated while still in the presence of the stimulus. **(B)** Simplified chemosensing signaling pathway, including activaiton of heterotrimeric G protein, Ras, PI3K, and PTEN in *Dictyostelium discoideum*. **(C)** Dynamics of chemoattractant concentration of chemoattractant, G protein activation, Ras activation, PIP_3_ production, and membrane-bound PTEN upon uniform stimulation.

Adaptation behavior can be characterized by the cellular responses to a homogeneously applied, persistent stimulation (uniform stimulation) (**Figure 1**). The cell response to this uniform stimulation reveals temporal features of adaptation. In *D. discoideum*, a uniform cAMP stimulation triggers a persistent G dissociation (G protein activation), while it induces transient, adaptive responses in many steps of the GPCR-mediated signaling pathway, such as activations of Ras and PI_3_K, membrane translocation of PTEN, and actin polymerization in *D. discoideum* (**Figures 1B**) (13, 15). These characteristics indicate that adaptation occurs downstream of heterotrimeric G protein. Many abstract models have been proposed to explain the dynamics of adaptation (14, 16, 17). They postulate that an increase in receptor occupancy activates two antagonistic signaling processes, namely, a rapid “excitation” that triggers cellular responses and a temporally delayed “inhibition” that terminates the responses and results in adaptation (6, 13, 14, 16–20). The central debate focuses on the spatial distribution and the activation mechanism of the inhibitor(s) that balances excitation to achieve temporal adaptation and spatial signal amplification for gradient sensing (6, 13, 14, 16, 19). One locally controlled inhibitory process has been revealed to be essential for gradient sensing in *D. discoideum* (17, 21, 22). Many excitatory components have been identified, while the inhibitors have just begun to be revealed (17, 23–26).

Here, we first review temporospatial features of chemoattractant GPCR-mediated Ras adaptation and then summarize recent findings on two membrane-targeting Ras inhibitors, C2GAP1 and CAPRI in *D. discoideum* and human neutrophils, respectively, that control the GPCR-mediated adaptation and sensitivity of cells (12, 25, 27). The functions of these two molecules suggest an evolutionarily conserved molecular mechanism by which eukaryotic cells gate the concentration range of chemoattractant gradients for chemotaxis.

## Chemoattractant GPCR-mediated Ras adaptation in chemotaxis of eukaryotic cells

*D. discoideum* has been used extensively as a eukaryotic model organism in the study of diverse cellular and developmental processes (28). Studies of cAMP-induced chemotaxis in *D. discoideum* have pioneered our understanding of how a eukaryotic cell senses extracellular chemoattractant stimuli for directional cell migration. Ras is a prototypical small GTPase and is a central regulator of cell growth, proliferation, and differentiation processes in every eukaryotic organism (29). Ras proteins are molecular switches that cycle between guanosine triphosphate (GTP)-bound active forms and guanosine diphosphate (GDP)-bound inactive forms. The conversion from stable, inactive GDP-bound forms to the active GTP-bound forms is stimulated by guanine nucleotide exchange-factors (GEFs). The conversion back from GTP-bound active forms to GDP-bound inactive forms is mediated by GTPase-activating proteins (GAPs). Large numbers of Ras, RasGEFs, and RasGAPs are encoded in *D. discoideum*: 15 Ras isoforms, 25 RasGEFs, and 17 RasGAPs (30, 31). Mammals express three Ras isoforms (K-, H-, and N-Ras). They are activated by three groups of RasGEFs: Sos (son of sevenless), RasGRFs (Ras guanine nucleotide-releasing factors), and RasGRPs (Ras guanine nucleotide-releasing proteins). They are deactivated by six groups of RasGAP proteins: p120GAP/RASA1, neurofibromin 1 (NF1), GAP1 family (GAP1m/RASA2, GAPIP4BP/RASA3, CAPRI/RASA4, and RASAL1), SynGAP family (SynGAP, DAB2ib, RASAL2, and RASAL3), Plexcin, and IQ-GAP (32, 33). Chemoattractants induce robust Ras activation in both *D. discoideum* and mammalian neutrophils (34, 35). Essential roles and activation mechanisms of Ras in chemotaxis were reported first in the model organism *D. discoideum* (36, 37) and later in mammalian neutrophils (38). Our current understanding of isoform-specific Ras functions is mostly based on the findings in cAMP-mediated chemotaxis in *D. discoideum* and is summarized here. cAMP binding to its GPCR, cAR1 (cAMP receptor 1), triggers the activation of three main Ras isoforms (RasC, RasG, and Rap1) that play essential roles in chemotaxis (34, 36, 39). However, cAMP stimulation triggers little Ras activation in cells deficient in Gβ(*gb−*), indicating that Gβγ plays a major role in inducing the initial transient Ras activation (40). Aimless (*aimless−*) is the first characterized RasGEF protein in *D. discoideum* that activates both RasC and RasG (34, 36, 39). In addition, RasG is also activated by GEFR, GEFF, and GflB (34, 41, 42). Reduced Ras activation is also observed in the cells deficient in GEFD (*gefD−*) and GEFM (*gefM−*), indicating their function in activating Ras (40). Several RasGAP proteins, including DdNF1, NF1, C2GAP1, IQGAPC, and RGBARG, are reported to deactivate RasC and RasG (25, 26, 43–45). IQGAPC and RGBARG deactivate RasG during phagocytosis or macropinocytosis at the stage of vegetative cell growth and will not be reviewed here (44, 45). Rap 1 is a member of Ras family in *D. discoideum* that plays a key role in adhesion during cell motility (46). cAMP-triggered transient Rap1 activation was first reported through Rap GEF GbpD in a RasG-dependent fashion (47, 48). Later, Gα2-binding GflB, which contains a RapGEF domain, directly activates Rap1 in a Gα2-dependent fashion in *Dictyostelium* (49). Interestingly, GflB also possess a RhoGAP domain and therefore balances Ras/Rap1/Rho activation during chemotaxis and cell migration (41, 49). Several GAP proteins, including RapGAP1, RapGAP3, and C2GAP1, deactivate Rap1 (25, 46, 50). Importantly, Ras activation is the initial known signaling event that displays adaptation behavior in the GPCR-mediated signaling pathways and is a key player in basal activity and excitability of a cell for cell migration in *D. discoideum* (37, 51, 52). In this review, we focus on reviewing the temporospatial profiles of Ras activation and adaptation in *D. discoideum* and then on the roles of C2GAP1 and CAPRI in GPCR-mediated Ras adaptation and sensitivity in both *D. discoideum* and human neutrophils.

### Temporospatial features of Ras activation and adaptation in chemoattractant-sensing *D. discoideum*

**Figure 2:**
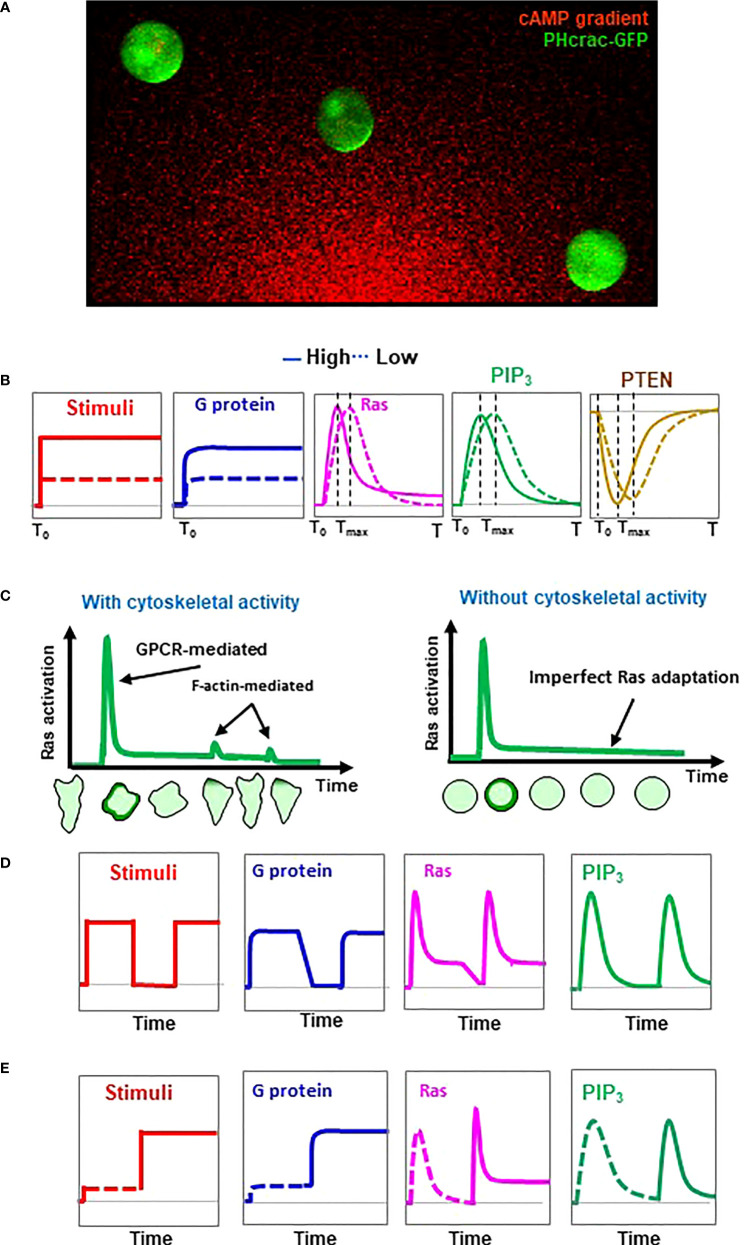
Concentration-dependent adaptation profiles of the GPCR-mediated signaling steps in chemosensing *D. discoideum* cells. **(A)** Demonstration of gradient sensing as an accumulation of PIP_3_ biosensor PH_Crac_-GFP (PH_Crac_-GFP crescents) in the fronts of the cells facing the source of the cAMP gradient. Cells expressing PIP_3_ biosensor PH_Crac_-GFP (green) were treated with actin polymeriztion inhibitor and exposed to a cAMP graident (red). Cells that lost the initial polarity and migration still sense cAMP gradient, indicating that gradient- sensing capabiltiy can be uncoupled from cell polarity and motility in *D. discoideum*. **(B)** Concentration-dependent dynamic activation of heterotrimeric G protein, Ras, PIP_3_ production, and membrane-bound PTEN in response to uniform stimulation of chemoattractant at a high or a low concentration. **(C)** Schemes show chemoattractant-induced Ras activation and adaptation with or without F-actin-based cytoskeleton activity. Representations of cells illustrate cells expressing active Ras probe RBD-GFP at the various time points upon uniform stimulation. **(D)** Activation profile of heterotrimeric G protein, Ras, PIP_3_ production, and membrane-bound PTEN in response to two identifical uniform stimulations. Uniform stimulation-induced transient translocation of RBD-GFP to the entire cell periphery and then accumulations at the protusion sites in the cell with cytoskeletal activity are shown beneath the graphs. **(E)** Activation profile of heterotrimeric G protein, Ras, PIP_3_ production, and membrane-bound PTEN in response to sequentially applied uniform stimulation at a low, then a high concentration.

Adaptation provides a fundamental mechanism for establishing an intracellular polarization in order to chemotax through a gradient with a large concentration range (53). *D. discoideum* provides a simplified cell system in which the gradient sensing can be uncoupled from cell polarity and cell motility (14). *D. discoideum* cells treated with actin polymerization inhibitors lose cellular polarity and motility and become spherical (**Figure 2**). These immobile, non-polarized cells maintain the ability to respond to chemoattractant stimuli and establish intracellular polarizations in a gradient as an accumulation of PIP_3_ as PH_Crac_-GFP crescents (PH_Crac_-GFP, a PIP_3_ biosensor) in the fronts (14). Using these immobile cells, the activation profile of several GPCR-mediated signaling events upon uniform stimulation at different concentrations was determined (**Figure 2**). Uniform cAMP stimulation triggers an instant, persistent dissociation (activation) of heterotrimeric G protein (22). A higher-concentration stimulation triggers a stronger constant activation of G proteins, indicating that the level of heterotrimeric G protein activation reflects the concentration of the stimulation. However, cAMP stimulation triggers a transient PIP_3_ production that is regulated mainly by two key enzymes, PI_3_K and PTEN (7, 54). Uniform cAMP stimulation triggers a transient PIP_3_ production by PI_3_K on the plasma membrane followed by a complete return to the pre-stimulus state, an adaptation behavior designated a perfect adaptation (22, 37). More importantly, higher-concentration stimuli trigger faster PIP_3_ production followed by adaptation in the cells (22). To quantify the temporal feature of adaptation, the time required for reaching a peak response was designated T_max_. A stronger stimulus result in a shorter T_max_ of PIP_3_ production. cAMP stimulation triggers a transient withdrawal of PTEN, i.e., a phosphatase dephosphorylates PIP_3_ to PIP_2_, on the plasma membrane. (7, 54, 55). The membrane localization of PTEN requires a PI(4,5)P_2_-binding motif at its N-terminus, but does not require PTEN phosphatase activity, the actin cytoskeleton, or the intracellular level of PI(3,4,5)P_3_. This reciprocal dynamics of PTEN, a transient withdrawal of PTEN and then return to the plasma membrane to the pre-stimulus state, suggests a perfect adaptation (17, 21). A stronger cAMP stimulus triggers a faster withdrawal and then a quicker return of PTEN to the plasma membrane. These results indicate that stronger stimuli not only trigger stronger activations of PI_3_K and a stronger PTEN response, but also initiate a stronger inhibitory mechanism by which to terminate these responses, thereby achieving adaptation.

Ras is a direct upstream activator of PI_3_K (37). A uniform cAMP stimulation triggers a transient Ras activation; thus, it becomes the first known adaptive response upstream of PI_3_K and PTEN in GPCR-mediated signaling pathways (**Figure 1**). Recent studies show that F-actin-based cytoskeleton plays important roles in Ras activation. For an example, F-actin-dependent activation of RasC through the Sca1/RasGEF/PP2A complex is crucial for refining the leading edge of a cell during chemotaxis (23). It has also been shown that GflB is stimulated by Gα2 in an F-actin-dependent fashion and plays an essential role in amplifying Gα2 signaling for Ras/Rap/Rho activation during gradient sensing and cell migration (41, 49). A close investigation of Ras activation at the subcellular level revealed that a uniform cAMP stimulation triggers a robust activation along the entire periphery of the cells, followed by a small reactivation which occurs at protrusion sites of the cells with an intact cytoskeleton (25) (**Figure 2**, left). In F-actin-free, immobile cells, uniform cAMP stimulation triggers only the initial transient Ras activation - a single phase of Ras activation - followed by an adaptation (**Figure 2**, right). These results indicate that the initial Ras activation is GPCR-induced, and the secondary reactivation of Ras occurs at and depends on the protrusions, a cellular structure that is closely related to the F-actin-based cytoskeletal activity. Computational simulations and experimental verification performed on the dose-dependent Ras activation gave contradictory results regarding the adaptive behavior of Ras signaling (18, 56, 57). Simultaneous monitoring of PIP_3_ production and Ras activation in the same cells provides three important conclusions on Ras adaptation (25). First, Ras activation and adaptation occur faster than those of PI_3_K, consistent with the fact that Ras is the direct activator of PI_3_K. Second, cells show a faster activation and adaptation of Ras and PIP_3_ production in response to a stronger stimulation. Third, cells display a transient Ras activation followed by a concentration-dependent imperfect adaptation, while they display a quicker production and a faster perfect adaptation of PIP_3_. A concentration-dependent imperfect adaptation means that in response to a low-concentration stimulus, cells display a transient Ras activation and then adapt to the pre-stimulus state; in response to a high-concentration stimulus, cells display a transient Ras activation, then adapt to a state in which the cells’ Ras activity state is greater than the pre-stimulus state, a process named imperfect adaptation (**Figure 2**). This concentration-dependent, imperfectly adaptive behavior of Ras activation has been further verified by two different types of two-dose experiments (58). One two-dose experiment is to apply an initial persistent stimulus to the cells, then remove the stimulus from the cells, and then apply a second, identical stimulus to the cells (**Figure 2**). In response to the sequentially applied identical stimuli, cells display a transient Ras activation and then adapt in response to the initial stimulus. The level of active Ras during the adaptation further decreases upon removal of the stimulus, demonstrating the imperfect Ras adaptation. When stimulated with a second identical stimulation, the cells show a transient Ras activation very similar to the initial activation. The other two-dose experiment is two-step stimulation: cells are stimulated with cAMP first at a low concentration, then at a high-concentration (**Figure 2**). In response to the first (low-concentration) stimulation, the level of active Ras in the adaptive cells returns to the pre-stimulated state after the initial transient activation. However, in response to the second (high-concentration) stimulation, the level of active Ras remains at a significantly higher level upon the second high-concentration stimulation after the second Ras activation. All of the above observations demonstrate that more Ras remains active after adaptation in response to a stronger stimulation. More importantly, imperfect Ras adaptation suggests an intracellular gradient of active Ras in gradient-sensing *D. discoideum* cells that might serve as an intracellular cue to activate PI_3_K and establish the PIP_3_ polarization.

### Spatial features of Ras activation and adaptation in gradient-sensing cells

**Figure 3:**
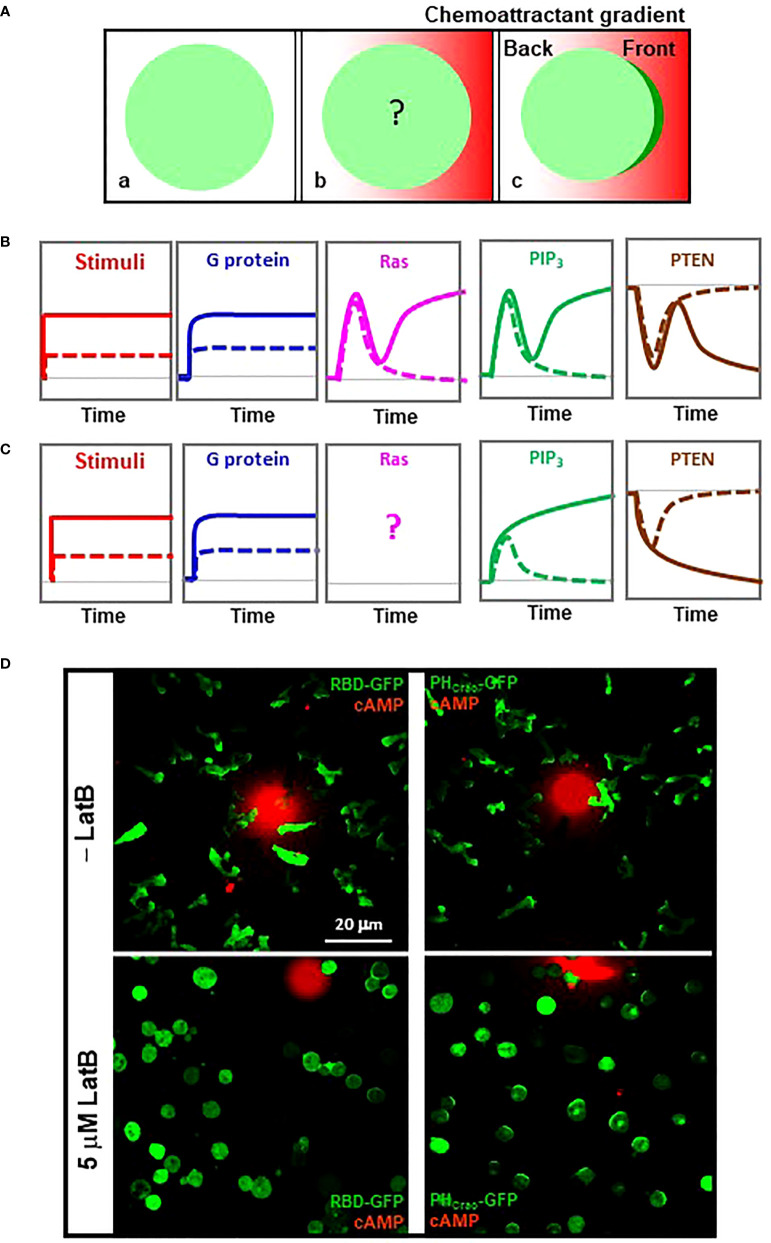
Establishment of an intracellular polarization in chemosensing *D. discoideum* cells experiencing a stable chemoattractant gradient. **(A)** Scheme shows how to monitor signaling events in the cells exposed to a stable chemoattractant gradient. a, a resting cells expressing a cytosolic protein (green); b, cell is stimulated by a chemoattractant gradient (red); c, cell in gradient sensing, in which the cell display an accumulation of the cytosolic protein in the front facing to the gradient. **(B)** Temporospatial dynamics of heterotrimeric G protein activation, Ras activation, PIP_3_ production, and membrane-bound PTEN in the front (solid colored line) and back (dotted colored line) of a *D. discoideum* cell upon exposure to a stable high-concentration cAMP gradient. Grey dotted lines along the bottom of each graph indicate the basal state. **(C)** Temporospatial dynamics of heterotrimeric G protein, Ras activation, PIP_3_ production, and PTEN translocation in the front (solid colored line) and back (dotted colored line) of a *D. discoideum* cell upon exposure to a stable low-concentration cAMP gradient. Grey dotted lines indicate the basal state. **(D)** Active Ras and PIP_3_ polarization in gradient-sensing *D. discoideum* cells with or without actin-based cytoskeleton. Cells expressing active Ras probe RBD-GFP (green) or PIP_3_ probe PH_Crac_-GFP (green) were treated with 5 μM latrunculin B in the lower panels. To visualize cAMP gradients, cAMP at the indicated concentrations was mixed with Alexa594 (red).

Eukaryotic cells distinguish a shallow difference of chemoattractant concentration between their front and back and establish an intracellular polarization for directional cell migration. F-actin free, immobile *D. discoideum* cells are still able to sense extracellular gradients (**Figure 2**) (14). Without the complication of the initial polarity and motility, immobile cells provide a simplified cell system in which to quantitatively measure signaling events in gradient-sensing cells. To understand the contribution of each signaling component in the processes of transducing and amplifying an exocellular gradient into an intracellular polarization, we abruptly exposed naive cells, which had not experienced chemoattractant, to a stable cAMP gradient (**Figure 3**). We monitored the differences in cAMP concentrations, G protein activation, PIP_3_ production, and PTEN dynamics between the front and the back of cells upon exposure to a steady gradient (**Figure 3**). Upon exposure to this stable gradient, the cells constantly experience a higher concentration of cAMP in the front than in the back, and this difference is accompanied by a persistently higher level of G protein activation in the front than in the back of the cells (22), indicating that the level of G protein activation reflects the local concentration of the gradient.

The responses of PIP_3_ production exemplify the process of gradient sensing in cells upon exposure to a steady gradient, a process in which each cell translates the extracellular gradient into an intracellular polarization that serves as an intracellular cue for directional cell migration (**Figure 3**). Upon exposure to a strong steep gradient, a cell displays a two-phase PIP_3_ production: an initial transient production around the cell membrane, followed by a second phase of PIP_3_ production only in the front of the cell, demonstrating the achievement of a PIP_3_ polarization (gradient sensing) (**Figure 3**) (22). The reciprocal dynamics of PTEN are also bi-phasic: an initial transient and asymmetrical withdrawal from the plasma membrane, followed by a second leave from the front (21). Upon exposure to a low-concentration gradient, a cell displays an altered PIP_3_ and PTEN dynamics (**Figure 3**). PIP_3_ is asymmetrically produced in both the front and the back of the cell, while the PIP_3_ in the back decreases and that in the front continuously accumulates to reach a steady level. PTEN asymmetrically withdraws from the plasma membrane at both the front and the back, and then more PTEN further withdraws from the front and return to the plasma membrane at the back (21). The different PIP_3_ and PTEN dynamics upon exposure to a high- or a low-concentration gradient suggest a concentration-dependent adaptation process during gradient sensing.

Ras dynamics have been characterized previously (40). In response to a strong 1 μM cAMP gradient, the temporospatial profile of Ras activation is also bi-phasic: an initial transient Ras activation around the cell membrane, followed by a second phase of Ras activation only in the front of the cell, demonstrating Ras adaptation and polarization to achieve signal amplification (**Figure 3**). Temporospatial Ras dynamics in response to a low-concentration gradient remain to be determined, although a concentration-dependent accumulation of Ras has been reported (40). Consistent with the above, we found that those immobile cells close to the sources of a strong gradient display a detectable accumulation of active Ras although chemotaxing cells always show clear accumulations of active Ras in the leading fronts (**Figure 3**, left panels), indicating that F-actin-based cytoskeleton provides positive feedback in Ras activation in chemotaxing cells. In contrast to Ras polarization, PIP_3_ crescent formation in the cells in gradients with a large concentration range exemplifies a cytoskeleton-independent, concentration-independent gradient-sensing capability of eukaryotic cells (**Figure 3**, right panels). The above indicates that a cell requires a steep, strong gradient to display a polarized Ras activation and that there is significant signal amplification from active Ras to PIP_3_ polarization during gradient sensing.

## RasGAP-mediated Ras adaptation in *D. discoideum* and neutrophils

Ras is deactivated by Ras GAPs to reach adaptation. In this section, we review the roles of two Ras GAP proteins, C2GAP1 and CAPRI, in Ras adaptation in *D. discoideum* and neutrophils, respectively.

### C2GAP1-mediated Ras adaptation in *D. discoideum*

*D. discoideum* encodes 17 RasGAP proteins (25). Single disruption of either *nf1* (*nf1^−^* or *axeB^−^*) or *ddnf1* (*ddnf1* ^−^ or *nfaA^−^*) results in enhanced Ras activity in the cells, consistent with the functions of these molecules as negative regulators of Ras activity (26, 43). Loss of NF1 (*axeB^−^*) enables *D. discoideum* cells to grow axenically in the culture medium by increasing fluid uptake through macropinocytosis (43), indicating that Ras functions in these cellular processes. In response to cAMP stimulation, *ddnf1* ^−^ cells display increased, prolonged activations of Ras and its effectors as well as impaired chemotaxis, indicating an essential role of DdNF1 in deactivating cAMP-induced Ras activation and chemotaxis (26). However, the molecular mechanism of DdNF1-mediated Ras adaptation remains unclear. We have previously demonstrated the involvement of a local inhibitory process that acts upstream of PI_3_K in gradient sensing (17, 21, 22). This motivated us to search for RasGAPs that target the plasma membrane. We identified C2GAP1, which contains a C2 domain that mediates the localization of its host proteins to the plasma membrane (7, 54, 55). We found that C2GAP1, the coding gene for which is *c2gapA*, is specifically expressed in the cAMP-sensing early developmental stage in *D. discoideum* (25), indicating its potential role in the cAMP-mediated chemotaxis.

**Figure 4:**
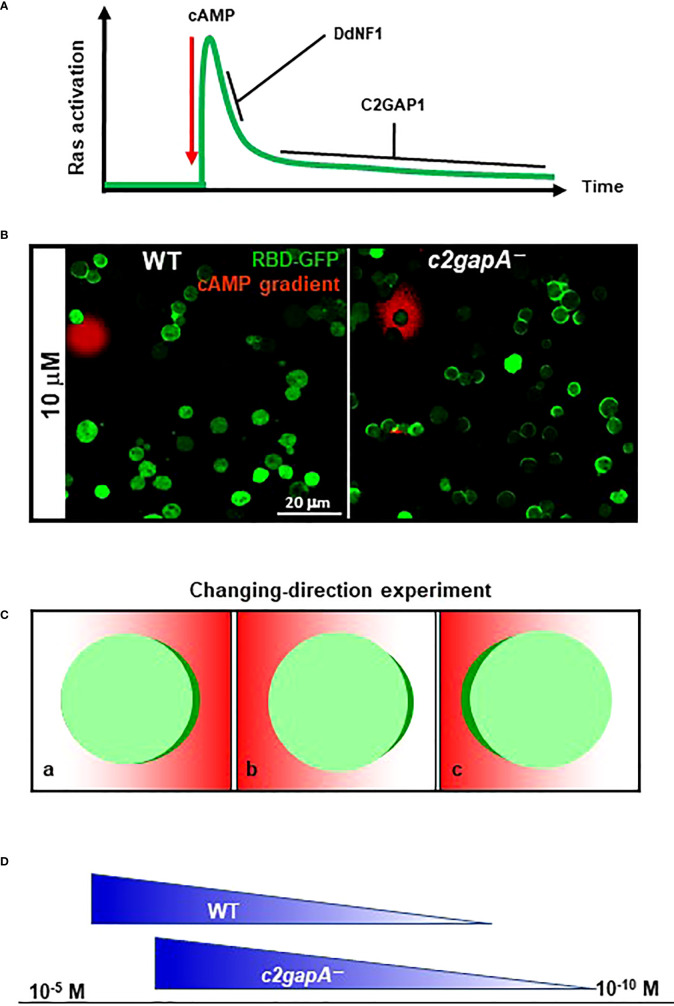
C2GAP1 in Ras adaptation, gradient sensing, and sensitivity of *D. discoideum* cells. **(A)** C2GAP1 plays a major role in persistent Ras adaptation after the initial Ras activation. Both DdNF1 and C2GAP1 are required for the adaptation process and act at different steps. DdNF1 is responsible for the quick turn-off of Ras activation at the initial step, and C2GAP1 plays a crucial role in persistent, long-term adaptation at the second step of the adaptation. **(B)** Increased Ras activation and polarization in *c2gapA−*cells in a cAMP gradient. Both WT and *c2gapA−* cells expressing active Ras probe RBD-GFP (green) were treated with 5 μM latrunculin in a cAMP gradient (red). **(C)** Scheme shows an experimental design changing the direction of a gradient. A cell expressing PIP_3_ probe PH_Crac_-GFP (green) is treated with actin polymerization inhibitor and displays accumulation of PH_Crac_-GFP (green) in a cAMP gradient (red) (a); upon changing the direction of the gradient to the opposite side of the cell (b), the cell accumulates PH_Crac_-GFP (polarization) facing the gradient (c). **(D)** Ras inhibitor increases the concentration range of chemoattractant in *D. discoideum*. The X-axis represents the concentration range of chemoattractant. In contrast with WT cells that chemotax in gradient with the concentraion range of 10^-9^ ~ 10^-5^ M, *c2gapA*−cells chemotax in the gradient with an upshifted concentration range of 10^-10^ ~ 10^-6^ M.

A cell that is deficient in Ras deactivator is expected to display increased Ras activation. *D. discoideum* cells deficient in *c2gapA* (*c2gapA^−^*) display significantly increased activation of Ras and its downstream effectors upon cAMP stimulation (25). However, there are differences in the non-adaptive behaviors between *c2gapA^−^* and *ddnf1^−^* cells. In response to uniform cAMP stimulation, an F-actin-free wild-type (WT) cell shows a transient Ras activation and then adapts, a typical single-phase activation profile of Ras, while *ddnf1* ^−^ cell exhibits a prolonged Ras activation (26). *c2gapA^-^* cells display the same initial, transient Ras activation as a WT cell does. *c2gapA−*cells fail to adapt persistently to the stimulation and display a second activation of Ras (25). Based on the Ras activation profiles in WT, *ddnf* ^-^ *−*, and *c2gapA−* cells, we suggest that the F-actin independent adaptation of Ras signaling includes two sequential steps: an initial, transient activation, followed by a persistent adaptation. Both DdNF1 and C2GAP1 are required for the adaptation process and act at different steps (**Figure 4**). DdNF1 appears to be responsible for the quick turn-off of Ras activation at the initial step, and C2GAP1 plays a crucial role in persistent, long-term inhibition at the second step of the adaptation. The F-actin-based cytoskeleton provides a positive or negative feedback regulation of Ras activation in chemotaxing cells (14, 23). F-actin-independent adaptation is involved in and contributes to the adaptation mechanism with the cytoskeletal activity.

Non-adaptive Ras activation in *c2gapA−* cells results in hyperactivation of downstream effectors, impaired gradient sensing, excessive polymerization of actin, and subsequent impaired chemotaxis. In a cAMP gradient, we found that immobile *c2gapA−* cells generate significantly increased accumulation of active Ras probe, RBD-GFP, in the fronts of the cells across the gradient; in contrast to WT cells, only those close to the source of gradient display accumulation of RBD-GFP in the front facing the gradient (**Figure 4**). Consistent with Ras activation, *c2gapA−*cells generate significantly broadened PH_Crac_-GFP crescents, manifesting an essential role of C2GAP1 in deactivating Ras to establish a sharp PIP_3_ polarization, a symbol of gradient-sensing machinery, in the cells. A key feature of sufficient gradient sensing is to enable a cell to promptly reorient its polarity to align with an unstable, direction-changing gradient (**Figure 4**). When the gradient changes, *c2gapA−*cells take a significantly longer time to correctly reestablish an intracellular polarization, if they are able to reestablish it at all (25), demonstrating an essential role of C2GAP1 in reorienting cell polarity toward a new gradient direction.

How a chemoattractant GPCR regulates inhibitors to achieve adaptation and gradient sensing of eukaryotic cell chemotaxis remains largely elusive. Two simple networks for receptor-induced adaptation have been proposed: an incoherent feedforward loop with a proportional node (IFFLP) and a negative feedback loop with a buffer node (NFBLB) (18, 20). The difference between IFFLP and NFBLB is the activation mechanism of the negative regulator to terminate the cell response to reach adaptation. In the IFFLP model, GPCR signaling *via* a component other than the product of the response activates the negative regulator, while in the NFBLB model, the response itself triggers the activation of the negative regulator (13, 18). Several models have proposed that the chemoattractant GPCR-induced inhibitions are likely IFFLP-type (14, 20, 21). The discovery of a local inhibition that acts upstream of PI_3_K in gradient sensing motivated us to investigate C2GAP1, an inhibitory protein for Ras adaptation (17, 21, 22). Interestingly, the temporospatial distribution of C2GAP1 in the cells closely follows and is similar to that of active Ras (25). However, C2GAP1 binds active or inactive Ras similarly, indicating that other factors, rather than the active state of Ras, are essential for its membrane targeting. Indeed, membrane translocation of C2GAP1 requires its C2 domain (25). The C2 domain often binds to Ca^2+^ and translocates to and further binds phospholipids in the plasma membrane (59, 60). Unexpectedly, the interaction between C2GAP1 and Ras is significantly reduced with the presence of high [Ca^2+^]. Consistent with the above, *iplA−*cells, which lack the IP_3_ receptor and triggers no [Ca^2+^] increase upon cAMP stimulation, display a prolonged membrane translocation of C2GAP1 (27), indicating that the GPCR-mediated calcium signaling negatively regulates the membrane translocation of C2GAP1. On the plasma membrane, C2GAP1 binds phospholipids, including the substrates and products of PI_3_K and PTEN (27, 37, 55, 61). However, C2GAP1 still translocates to the plasma membrane in cells treated with the PI_3_K inhibitors (25), indicating the products of PI_3_K are not essential for its membrane targeting and PIP_3_ might provide another layer of regulation for the recruitment of C2GAP1. Thus, membrane targeting of C2GAP1 is consistent with an IFFLP-based network for GPCR-induced Ras adaptation.

### CAPRI controls Ras adaptation in human neutrophils

Neutrophils also use GPCR-mediated signaling pathways to sense and move toward gradients of diverse chemoattractants. Briefly, chemoattractants engage with their GPCRs and activate inhibitory heterotrimeric G protein of Gβγ and Gαi (62). Both Gαi-GTP and Gβγ activate several downstream effectors, including PI3Kγ and PLCβ2/3 (63–65). Diverse chemoattractants, such as fMLP, interleukin-8 (IL-8, ligand of CXCR1 and CXCR2), and complement component 5a (C5a, ligand of C5AR1), also trigger robust Ras activation in neutrophils (35). Both active Ras and Gβγ directly activate PI3Kγ, a key enzyme in neutrophils, to phosphorylate PIP_2_ to PIP_3_ (38, 66–68). Chemoattractant-induced PIP_3_ production is also regulated by PTEN in mammalian neutrophils. In contrast to PM-localizing PTEN in *D. discoideum*, mammalian PTEN localizes in the cytoplasm in resting neutrophils (69). Chemoattractant stimulation induces PM translocation of PTEN, where PTEN dephosphorylates PIP_3_ to PIP_2_ (70). Chemoattractant-induced PI3Kγ activation and PTEN PM translocation together mediate a transient PIP_3_ production, which plays an essential role in neutrophil chemotaxis (70, 71). Human neutrophil-like (HL60) cells, a model cell system for mammalian neutrophils, display a transient Ras activation and an F-actin-based Ras reactivation (oscillation) in response to uniform stimulation and accumulate active Ras at the leading edge during chemotaxis, as does *Dictyostelium* (26, 34, 72). Ras signaling, as a direct regulator of PI3Kγ, PKB/AKT, and ROS production, plays an important role in neutrophil chemotaxis and neutrophil biology (38, 71).

Neutrophils can also sense and chemotax through chemoattractant gradients with a large concentration range (10^-5^~10^-9^ M fMLP and 10^-5^~10^-9^ M IL8, respectively) (12). To accurately navigate through a chemoattractant gradient, neutrophils also employ adaptation as one fundamental mechanism. Diverse chemoattractants trigger transient, adaptive Ras activation in mammalian neutrophils (35). Neutrophils highly express N-Ras and low H- and K-Ras and two classes of RasGEFs: RasGRP4 and Sos1/2 (12, 32, 38, 68, 73). Chemoattractant stimulation triggers a significantly reduced Ras activation in the mouse neutrophils deficient in RasGRP4, indicating a major role of RasGRP4 in chemoattractant-induced Ras activation in neutrophils (38), while it has been recently shown that Sos1/2 plays a major role in priming mouse neutrophils to increase responsiveness during inflammation (73). Human neutrophils highly express p120GAPs and CAPRI (74–76), while murine neutrophils also express RASAL1 and RASAL2 (12). Interestingly, inhibition of p120GAP leads to Ras activation in human neutrophils (76), suggesting its GAP activity in neutrophils. However, the role of p120GAP in chemoattractant-induced Ras activation remains unknown. Both mammalian neutrophils and HL60 cells highly express CAPRI, a calcium-promoted Ras inactivator which is a previously characterized Ras GAP protein in T-cell, HEK293, CHO, and COS-7 cells (12, 77–82). Recently, it was revealed that CAPRI mediates chemoattractant-triggered Ras adaptation and chemotaxis in HL60 cells (12). CAPRI localizes in the leading front of chemotaxing cells and translocates to the plasma membrane upon stimulation with diverse chemoattractants, just like C2GAP1 in *D. discoideum* (25). Chemoattractant stimulation promotes the association between CAPRI and N-Ras. *capri^kd^* cells, in which CAPRI expression is stably knocked down, exhibit increased, non-adaptive Ras in response to chemoattractant stimulation (12), demonstrating a major role of CAPRI in GPCR-mediated Ras adaptation in human neutrophils. The subsequent increased activation of Ras effectors, including AKT, Erk42/44, GSK3α/3β, and cofilin, leads to excessive actin polymerization and adhesion, and impaired chemotaxis.

Membrane targeting is required for RasGAPs to deactivate Ras. Several factors of GPCR signaling affect membrane recruitment of CAPRI. CAPRI was first characterized by the calcium dependency of its membrane translocation (79). Consistent with this, CAPRI mutant without the calcium-binding C2 domain does not translocate to the plasma membrane upon chemoattractant stimulation, indicating that calcium binding is required for CAPRI membrane targeting during chemotaxis (12). PH domains of the GAP1 family bind PI(3,4,5)P_3_ on the plasma membrane (83). CAPRI mutant without the PH domains shows significantly decreased membrane translocation, indicating that the GPCR-triggered PIP_3_ production plays a role in recruiting CAPRI to the plasma membrane. However, the GAP-inactive mutant of CAPRI still translocates to the plasma membrane just as wild-type (WT) CAPRI does, indicating that the GAP activity of CAPRI plays no role in its membrane targeting. CAPRI colocalizes with active Ras in the chemotaxing cells; however, CAPRI translocates to the plasma membrane in the cells without active Ras, indicating that the active state of Ras is not required for membrane recruitment of CAPRI. It has been previously reported that CAPRI interacts with Rho GTPase cdc42 and Rac1 in macrophages (75). CAPRI also colocalizes with F-actin in the leading edge of chemotaxing cells where Rac1 is enriched, suggesting an Rac1/F-actin-mediated recruitment of CAPRI. In conclusion, the GPCR-mediated calcium signaling and phospholipids on the plasma membrane, but not the active state of Ras or the GAP activity of CAPRI, play a major role in membrane targeting of CAPRI.

Multiple Ras GAPs might be involved in Ras adaptation in neutrophils. *capri^kd^* cells display nonadaptive Ras/Rap1 activation and excessive actin polymerization in response to fMLP stimuli at saturating concentrations. However, in response to fMLP stimuli at low concentrations (either 0.1 nM or 10 nM), *capri^kd^* cells display transient Ras/Rap1 activation and actin polymerization stimulation, indicating the involvement of other Ras GAPs for Ras adaptation. In fact, neutrophils express multiple Ras GAPs, such as RASALs in addition to CAPRI. RASALs are members of the GAP1 family (83). The coordination of CAPRI and RASAL for Ras signaling has been previously reported (78). Recently, RASALs have also been indicated to be involved in cell migration (84). p120 GAP, absent in HL60 cells, is present in both human and mouse neutrophils (85) and plays a role in the migration of mouse neutrophils (86, 87). While a major role of CAPRI in mediating GPCR-mediated Ras adaptation has been revealed in HL60 cells, it is also crucial to determine the potential roles of other RasGAP proteins and CAPRI in Ras adaptation and chemotaxis in primary mammalian neutrophils.

## RasGAP-mediated sensitivity of *D. discoideum* and human neutrophils

Little connection has been made between GPCR-mediated adaptation and the sensitivity of eukaryotic cells. It has been speculated that cells constantly deactivate Ras to maintain low level of the basal Ras activity in the resting state (32). In agreement with this speculation, we found that both C2GAP1 and CAPRI localize on the plasma membrane in the unstimulated, resting cells (12, 25). Consistent with this localization on the plasma membrane, both *c2gapA−*and *capri^kd^* cells display increased basal Ras activity in the resting cells, indicating the role of C2GAP1 and CAPRI in deactivating Ras to maintain a low basal activity of the cells. Interestingly, both cells spread faster morphologically and have very active protrusion formation. Both *c2gapA−*and *capri^kd^* cells are more sensitive to chemoattractant stimuli and show improved chemotaxis in low- or subsensitive-concentration gradients while displaying impaired chemotaxis in response to high-concentration gradients (12, 27), demonstrating that *c2gapA−*and *capri^kd^* cells display an upshifted concentration range of chemoattractant gradient for chemotaxis. Hence, *Dictyostelium* and neutrophils employ RasGAP proteins to control GPCR-mediated adaptation and cell sensitivity and consequently gate a chemoattractant concentration range for chemotaxis. In addition, similar factors are involved in the recruitment of C2GAP1 and CAPRI to the plasma membrane. Both molecules target the plasma membrane independent of the active state of Ras and RasGAP activity (12, 25). Both bind phospholipids of PI_3_K activation on the plasma membrane (27, 79, 83). Both bind F-actin but do not rely on F-actin for their membrane targeting (25, 75). Interestingly, [Ca^2+^] plays roles in the membrane targeting of both proteins. In addition, increased basal activity has also been reported in the other GAP-deficient cells, such as *axeB^−^* and *ddnf1^−^* (26, 43). All of the above indicate that membrane targeting of Ras GAP proteins is a general mechanism by which eukaryotic cells control the GPCR-mediated adaptation and the basal sensitivity of the cells.

Adaptation occurs at many steps of GPCR-regulated signaling pathways. The factors modulating basal cell sensitivity largely remain elusive. Is employing the same molecule to control both adaptation and cell sensitivity a general mechanism for chemoattractant sensing? If not, what are the other strategies? The role of Rap in chemotaxis might provide some interesting insight. Chemoattractants also trigger a transient, adaptive Rap activation, which induces cellular adhesion during migration in both *Dictyostelium* and neutrophils. Chemoattractants promote the interaction of C2GAP1 and CAPRI with Rap protein in *D. discoideum* and human leukocytes. Both *c2gapA−*and *capri^kd^* cells are flatter, a morphological signature of hyper Rap activity. In response to chemoattractant stimulation, both cells display increased Rap activation, demonstrating their function as RapGAP proteins. It has been reported that *Dictyostelium* cells use “switching” molecules that have RasGAP domains along with other domains to balance Ras/RAP or Ras/Rho signaling and might consequently alter the sensitivity of a cell through Ras-based basal activity (44, 49). In mammalian cells, it has been shown that CAPRI functions as a Rap GAP to inhibit Rap1 function in adhesion in T cells (80). Interestingly, it has been shown that CAPRI switches its activity to be either RasGAP or RapGAP to modulate Ras/Rap signaling in a cell (77). Nonetheless, it remains largely unclear whether Rap proteins are directly or indirectly involved in regulating basal activity and chemotaxis in eukaryotic cells. Identification and characterization of other inhibitors of GPCR signaling for adaptation or of molecules involved in modulating cell sensitivity will shed new light on how eukaryotic cells detect and respond to chemoattractants.

## Closing remarks

Recruitment of leukocytes to the sites of inflammation needs to be meticulously regulated during immune responses. Both insufficient and excessive recruitment and subsequent activation of leukocytes can cause diverse diseases. Ras plays a central role in the chemotaxis of *D. discoideum* and neutrophils and in basal cell activity. In this review, we have summarized the functions of two RasGAP proteins in *D. discoideum* and human neutrophils that deactivate active Ras to lower cell sensitivity and achieve GPCR-mediated adaptation. Their functions suggest an evolutionarily conserved mechanism by which eukaryotic cells use Ras inhibitors to gate the concentration range of chemoattractants for chemotaxis. Adaptation occurs at many steps of GPCR-regulated signaling pathways. In addition, the factors modulating basal cell sensitivity largely remain elusive. Identification and characterization of other inhibitors of GPCR signaling for adaptation will shed new light on how eukaryotic cells detect and respond to chemoattractants.

## Author contributions

XX wrote the manuscript and XX and TJ edited the manuscript. All authors contributed to the article and approved the submitted version.

## Funding

This work was supported by the intramural fund of the National Institute of Allergy and Infectious Diseases, National Institutes of Health.

## Conflict of interest

The authors declare that the research was conducted in the absence of any commercial or financial relationships that could be construed as a potential conflict of interest.

## Publisher’s note

All claims expressed in this article are solely those of the authors and do not necessarily represent those of their affiliated organizations, or those of the publisher, the editors and the reviewers. Any product that may be evaluated in this article, or claim that may be made by its manufacturer, is not guaranteed or endorsed by the publisher.
